# Anti-Inflammatory Triterpenoids from the *Caulophyllum robustum* Maximin LPS-Stimulated RAW264.7 Cells

**DOI:** 10.3390/molecules23051149

**Published:** 2018-05-11

**Authors:** Bin-Hua Qin, Xin-Qiao Liu, Qiao-Yu Yuan, Jing Wang, Hai-Yan Han

**Affiliations:** 1 School of Pharmaceutical Sciences, South-Central University for Nationalities, Wuhan 430074, China; binhuawuhan@163.com (B.-H.Q.); wjingwuhan@126.com (J.W.); 15827253179@163.com (H.-Y.H.); 2 Department of Bioengineering, Wuhan Polytechnic, Wuhan 430074, China; yqyscuec@163.com

**Keywords:** *Caulophyllum robustum*, triterpene derivatives, anti-inflammatory

## Abstract

*Caulophyllum robustum* Maxim is widely distributed in China and used as a traditional herbal medicine to induce childbirth, ease the pain of labor, rectify delayed or irregular menstruation, alleviate heavy bleeding and pain during menstruation, and treat external injuries and irregular menses. According to our detailed chemical investigation, three new triterpene derivatives (**1**–**3**), together with seven known compounds, were isolated from the root and rhizome of *C. robustum* Maxim. Their structures were elucidated by 1D- and 2D-NMR spectroscopic analysis and physio-chemical methods. They were identified as (**1**) 23-hydroxy-3,19-dioxo-olean-12-en-28-oic-acid; (**2**) 23-hydroxy-3,11-dioxo-olean-12-en-28-oic acid; and (**3**) 16*α*,23-dihydroxy-3-oxo-olean-12-en-28-oic acid. Compounds (**1**–**10**) inhibited the LPS-activated NO production in RAW264.7 cells. Furthermore, the anti-inflammatory characteristics of these compounds were confirmed on the basis of decreases in iNOS and NF-κB protein expression in RAW264.7 cells.

## 1. Introduction

**Figure 1:**
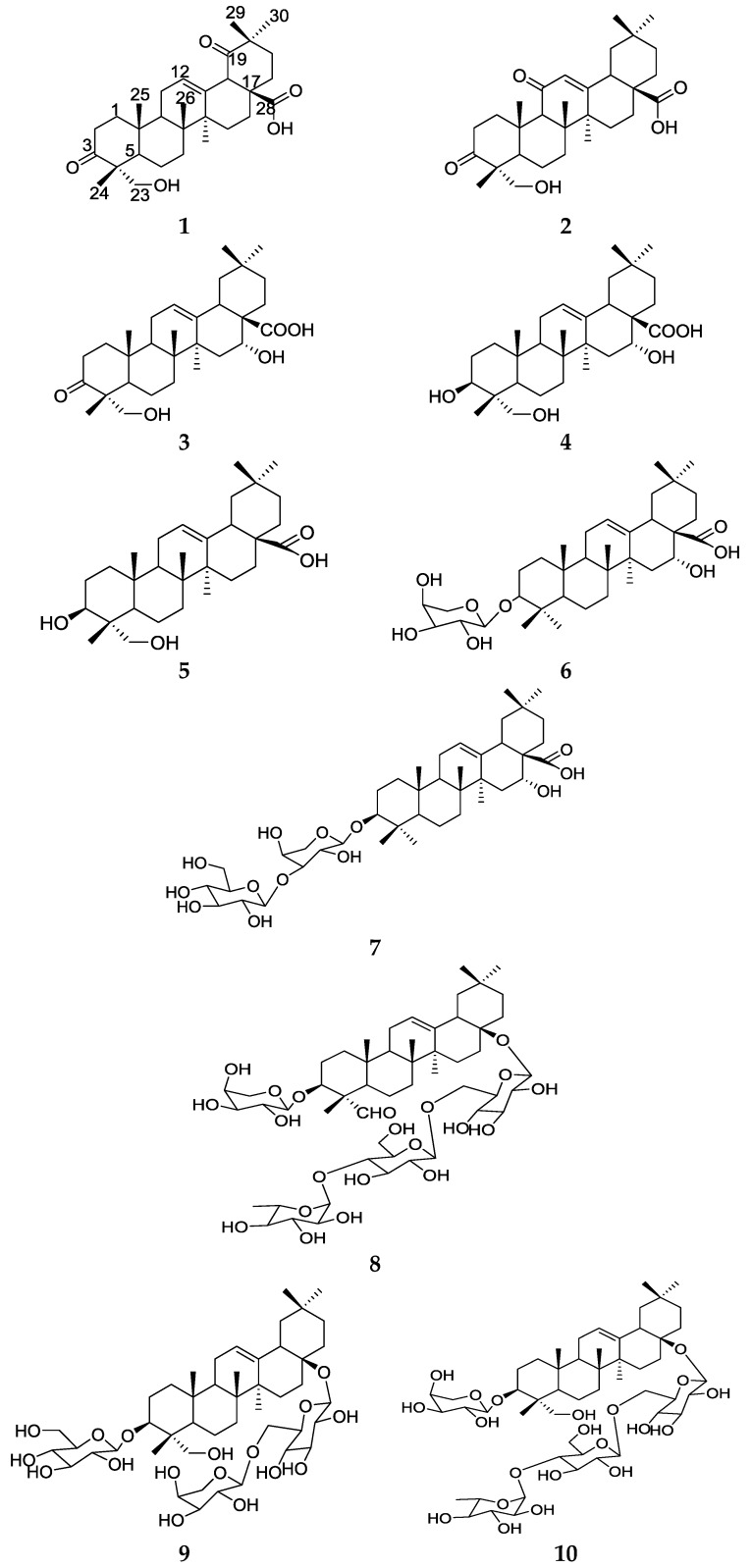
Chemical structures of triterpene derivatives **1**–**10**.

*Caulophyllum robustum* is a perennial herb in the family Berberidaceae, whichis well known as *Hong Mao Qi* in Chinese [1]. It is distributed in the northeast Tibet, Hubei provinces of China, native communities of North America, Its root and rhizome have beenused to induce childbirth, ease the pain of labor, rectify delayed or irregular menstruation, alleviate heavy bleeding and pain during menstruation [2]. Modern pharmacological studies have demonstrated that alkaloids and triterpenoidsaponins are responsible for its major biological function as an anti-inflammatory [3]. As part of our effort to search for novel triterpene from *C. robustum* Maxim, we report here the isolation and structure determination of the three new terpenoids: (**1**) 23-hydroxy-3,19-dioxo-olean-12-en-28-oic-acid; (**2**) 23-hydroxy-3,11-dioxo-olean-12-en-28-oic acid; and (**3**) 16*α*,23-dihydroxy-3-oxo-olean-12-en-28-oic acid (Figure 1). Together with this, we also report seven known other compounds: (**4**) collinsonin; (**5**) hederagenin; (**6**) echinocystic acid 3-*O*-*α-*l-arabinopyranoside; (**7**) saponin PE; (**8**) 3-*O*-*α-*l-arabinopyranosylgypsogenin-28-*O*-*α-*l-rhamnopyranosyl-(1-4)-*β*-d-glucopyranosyl-(1-6)-*β*-d-glucopyranosyl ester; (**9**) 3-*O*-*β*-d-glucopyranosylgypsogenin-28-*O*-*α-*l-arabinopyranosyl-(1-6)-*β*-d-glucopyranosyl ester, (**10**) 3-*O-α-*l-arabinopyransylgypsogenin-28-*O*-*α*-l-rhamnopyranosyl-(1-4)-*β*-d-glucopyranosyl-(1-6)-*β*-d-glucopyranosylester.

## 2. Results and Discussion

### 2.1. Structure Elucidation

**Figure 2:**
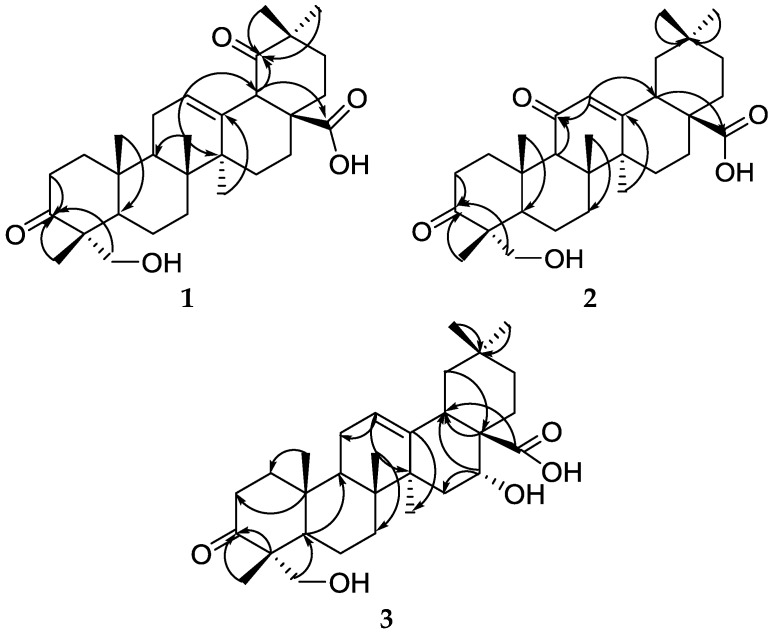
Key HMBC correlations of triterpenoids **1**–**3**.

The structures of the new compounds (**1**–**3**) were elucidated on the basis of extensive NMR spectroscopic analysis, including a series of 2D-NMR experiments (Figure 2) (HSQC, HMBC), and mass spectrometry data. The known compounds (**4**–**10**) were identified by comparison of their experimental spectral data with literature data [4,5,6,7,8,9,10].

Compound **1** was obtained as a white, amorphous powder. The molecular formula was determined to be C_30_H_44_O_5_ from the pseudomolecular ion peak [M − H]^−^ at *m*/*z* 483.3117 (calculated 483.3110) in the HR-ESI-MS. The ^1^H-NMR spectrum revealed six methyl group signals at *δ* (ppm) 0.86 (3H, s, H-24), 1.05 (3H, s, H-25), 0.85 (3H, s, H-26), 1.15 (3H, s, H-27), 0.95 (3H, s, H-28), and 1.21 (3H, s, H-30). It also revealed an olefinic proton at *δ* (ppm) 5.41 (1H, t, *J =* 3.0 Hz), which were characteristic of triterpene with the Δ^12^ oleanane skeleton [11], The ^1^H-NMR spectrum also showed signals for an oxymethylene groups at *δ*_H_ 3.56 (1H, d, *J* = 11.5 Hz, H-23a), 3.28 (1H, d, *J* = 11.5 Hz, H-23b). The ^13^C-NMR and DEPT (135° and 90°) spectra exhibited 30 carbon signals, assignable to sixmethyls, ten methylene, four methine, and ten quaternary carbons. The ^13^C-NMR spectrum (Table 1) showed signals of two carbonyls (*δ*_C_ 219.8 (C-3) and 216.2 (C-19)), a carboxy (*δ*_C_ 178.2, C-28), and an oxymethylene (*δ*_C_ 68.2 (C-23)), which was similar to those of 23-hydroxy-3-oxolean-12-en-28-oic acid [12] except for the presence of a carbonyl group in **1**. In the HMBC spectrum, the correlations between H-29 (*δ*_H_ 0.95), H-30 (*δ*_H_ 1.21), H-18 (*δ*_H_ 2.79, d, *J* = 14.0 Hz) and the carbonyl group (*δ*_C_ 216.2) reveled that this carbonyl group was located at C-19.andthe correlations between H-24 (*δ*_H_ 0.86), H-23 (*δ*_H_ 3.56, 3.28) and the carbonyl group (*δ*_C_ 219.8) reveled that this carbonyl group was located at C-3.On the basis of the above analyses, the structure of compound **1** was established as 23-hydroxy-3,19-dioxo-olean-12-en-28-oic acid.

Compound **2** was obtained as a white, amorphous powder. The molecular formula was determined to be C_30_H_44_O_5_ from the pseudomolecular ion peak [M − H]^−^ at *m*/*z* 483.3118 (calculated 483.3110) in the HR-ESI-MS. The ^1^H and ^13^C-NMR data (Table 2) of 2 were similar to those of compound **1**. Careful comparison of the NMR data between compound **1** and **2** indicated that both compounds possessed the same carbon skeleton but had a difference in the location of one carboxy group (*δ*_C_ 200.0). In the HMBC spectrum, the correlations between the H-12 (*δ*_H_ 5.64), H-9 (*δ*_H_ 2.43) andthe carbonyl group (*δ*_C_ 200.0) reveled that this carbonyl group was located at C-11, which could be further confirmed by the deshielding effect on the olefinic carbon (*δ*_C_ 169.4) in beta position withrespect to the carbonyl (C-11). Thus, compound **2** was a new compound, named 23-hydroxy-3,11-dioxo-olean-12-en-28-oic acid.

Compound **3** was obtained as a white, amorphous powder. The molecular formula was determined to be C_30_H_44_O_5_ from the pseudomolecular ion peak [M + H]^−^ at *m*/*z* 485.3271 (calculated 485.3267) in the HR-ESI-MS. The NMR data of 3 (Table 3) were similar to that of caulophyllogenin [13], except for the presence of a carbonyl group instead of a methylenoxy in 3. The HMBC spectrum, the correlations between the *δ*_H_ 3.53 (1H, d, *J* = 11.5 Hz, H-23a), 3.27 (1H, d, *J* = 11.5 Hz, H-23b), *δ*_H_ 0.85 (3H, s, H-24) and the carbonyl group (*δ*_C_ 220.0), indicated that this carbonyl group was located at C-3.The ROE correlations of H-16 (*δ*_H_ 4.41) with H-26 (*δ*_H_ 0.81) suggested H-16 on the same side (*β*-oriented), While 16-OH on the other side (*α*-oriented). On the basis of the above analyses and NMR spectroscopic data (Table 3), the structure of compound **3** was established as 16*α*,23-dihydroxy-3-oxo-olean-12-en-28-oic acid.

### 2.2. Biological Activity Assays

**Figure 3:**
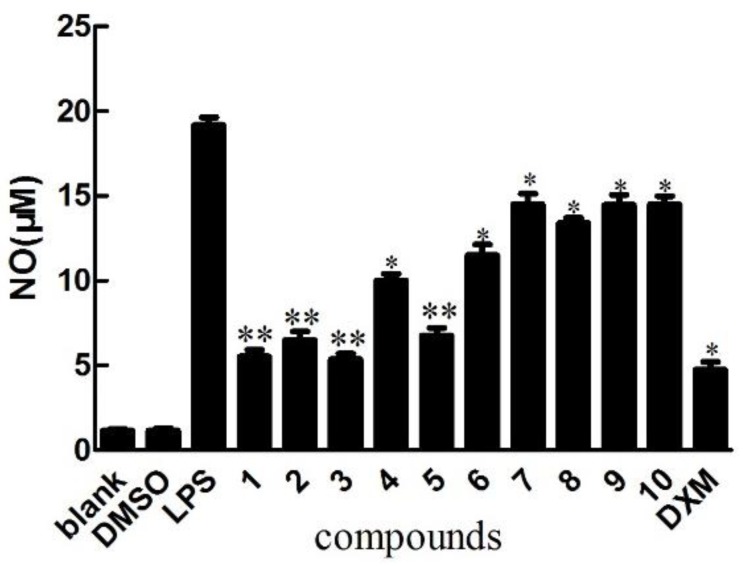
Anti-inflammatory effects of the isolated compounds, inhibition of LPS-induced NO production in RAW274.7 mouse macrophages. The cells were pre-treated with concentrations (25 μM) of compounds (**1**–**10**) or DXM (apositive control, 10 μM) for 1 h, then stimulated with LPS (5 μg/mL) for 24 h. Nitrite levels, which reflect NO levels in culture media, were measured using Griess assays. The data shows the mean ± SD of three independent experiments. ** *p* < 0.01 and * *p* < 0.05compared to the LPS-treated values.

**Figure 4:**
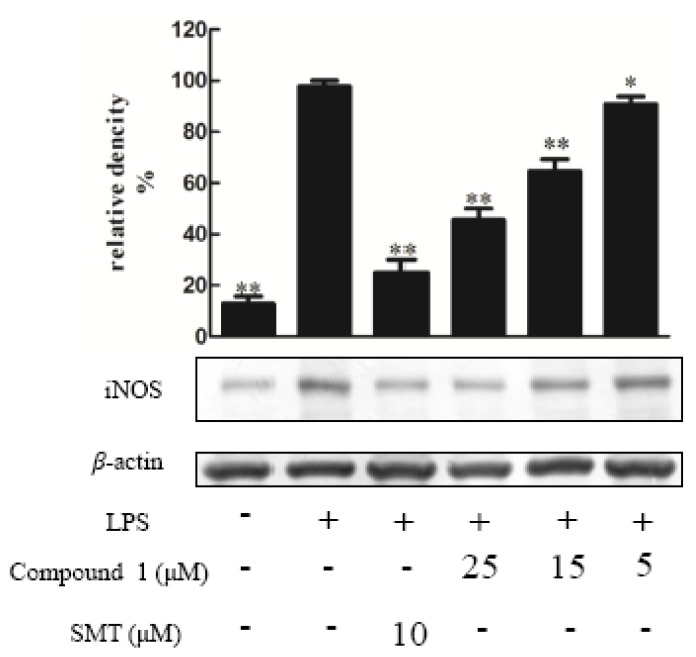
Effect of compound **1** on the expression of proteins associated with inhibition of iNOS in LPS-stimulated RAW274.7 mouse macrophages. The cells were pre-treated with different concentrations or SMT (an iNOS inhibitor, 10 μM) for 1 h, then stimulated with LPS (5 μg/mL) for 24 h. The expression of the iNOS protein was determined by Western blotting. The data shows the mean ± SD of three independent experiments performed in triplicates. ** *p* < 0.01 and * p < 0.05 compared to the LPS-treated values.

**Figure 5:**
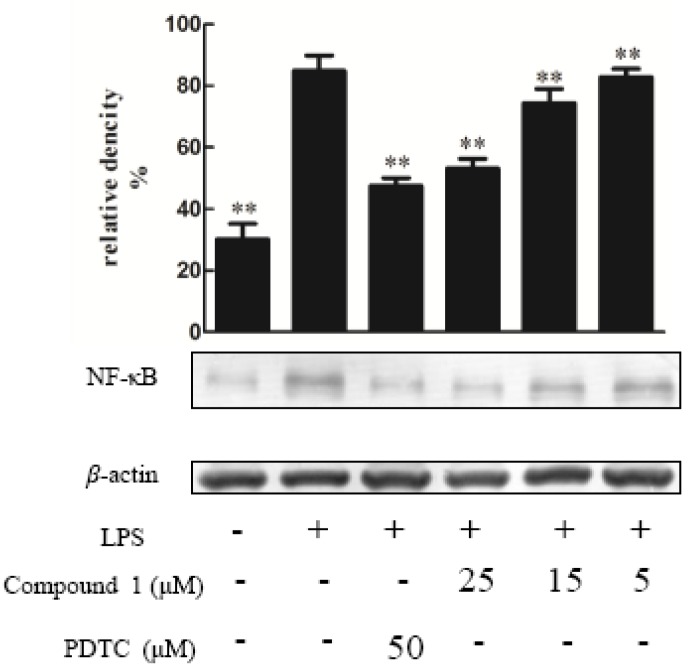
The effect of compound **1** on the expression of proteins associated with the inhibition of NF-κB in LPS-stimulated RAW274.7 mouse macrophages. The cells were pre-treated with different concentrations or PDTC (an NF-κB inhibitor, 10 μM) for 1 h, then stimulated with LPS (5 μg/mL) for 24 h. The expression of the NF-κB protein was determined by Western blotting. The data show the mean ± SD of three independent experiments performed in triplicates. ** *p* < 0.01 compared to the LPS-treated values.

**Figure 6:**
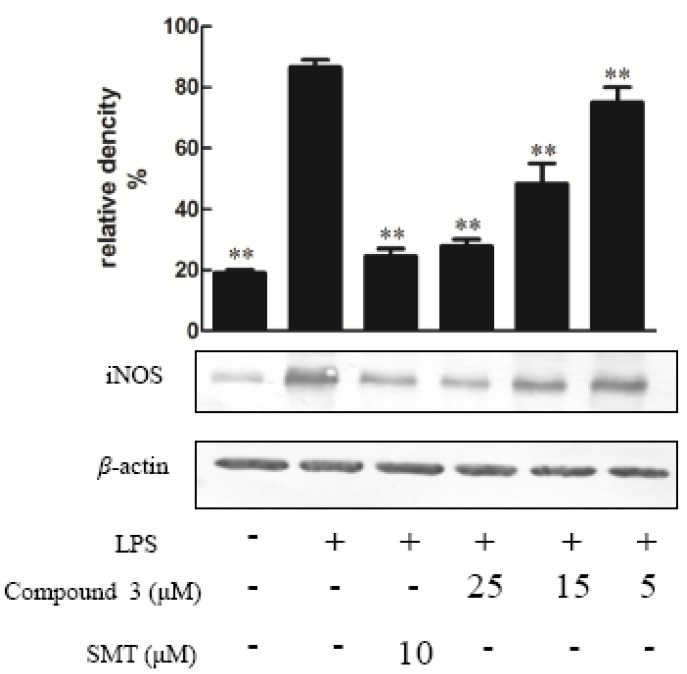
The effect of compound **3** on the expression of proteins associated with the inhibition of iNOS in LPS-stimulated RAW274.7 mouse macrophages. The cells were pre-treated with different concentrations or SMT (an iNOS inhibitor, 10 μM) for 1 h, then stimulated with LPS (5 μg/mL) for 24 h. The expression of the iNOS protein was determined by Western blotting. The data shows the mean ± SD of three independent experiments performed in triplicates. ** *p* < 0.01 compared to the LPS-treated values.

**Figure 7:**
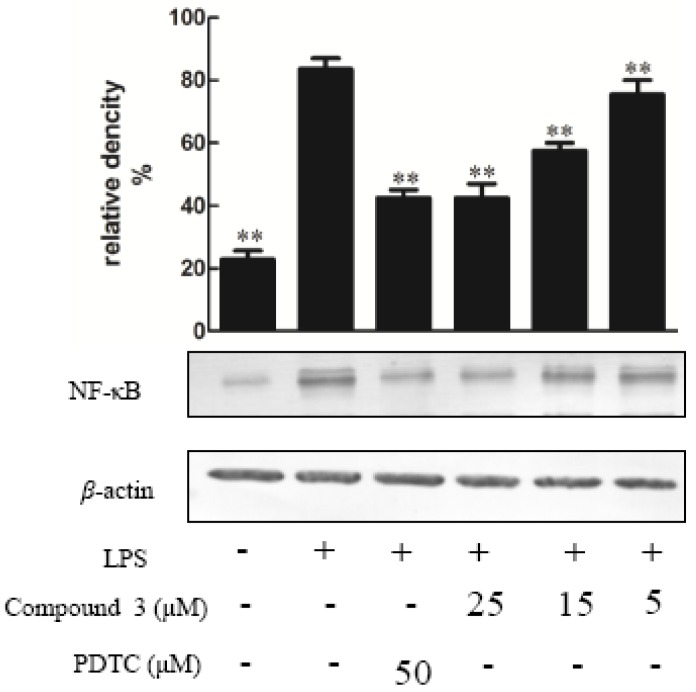
The effect of compound **3** on the expression of proteins associated with the inhibition of NF-κB in LPS-stimulated RAW274.7 mouse macrophages. The cells were pre-treated with different concentrations or PDTC (an NF-κB inhibitor, 10 μM) for 1 h, then stimulated with LPS (5 μg/mL) for 24 h. The expression of NF-κB protein was determined by Western blotting. The data show the mean ± SD of three independent experiments performed in triplicates. ** p < 0.01 compared to the LPS-treated values.

Compounds **1**–**10** were tested for their ability to inhibit NO production in LPS-activated RAW264.7 macrophages as a measure of their anti-inflammatory effects, apositive control was DXMwith the value of 10 μM. The result indicated that the NO production was decreased by the presence of compound **1**–**5** with values of 25 μΜ [14] (Figure 3), since NO was made by catalytic synthesis of iNOS. NF-κB is a transcriptional factor that acts as an important mediator of the immune response and controls the expression of several proteins involved in inflammation, such as iNOS and cyclooxygenase-2 (COX-2) [15]. To further confirm the anti-inflammatory properties of 1 and 3, we investigated their effects on the protein expressions of NF-κB and iNOS in LPS-induced RAW264.7 macrophage cells using Western blotting; these proteins are involved in the pathogenesis of chronic inflammatory diseases [16]. Consistent with their inhibitory activity toward NO, compounds **1** and **3** inhibited the induction of iNOS and NF-κB protein expression in a dose-dependent manner [11] (Figure 4, Figure 5, Figure 6 and Figure 7). Moreover, the housekeeping protein *β*-actin was not changed by the presence of compounds **1** and **3** at the same concentration.

## 3. Materials and Methods

### 3.1. General

Optical rotations were recorded on an A25700-T digital polarimeter (RUDOLPH, Hackettstown, NJ, USA). IR spectra were obtained using a Thermo Tensor Nicolet-6700 spectrometer (Thermo Optics, Inc., Billerica, MA, USA) with KBr pellets, and 1D and 2D NMR spectra were recorded on a Bruker DRX-600 instrument (600 MHz for ^1^H and 150 MHz for ^13^C) with TMS as an internal standard, the deuterated solvent used to solubilize the samples were CD_3_OD and CDCl_3_.HR-ESI-MS was recorded on an UPLC-Q Exactive MS system (Thermo Fisher, Santa Clara, CA, USA). Silica gel (200–300 mesh (Qingdao Haiyang Chemical Co., Ltd., Qingdao, China) and Sephadex LH-20 (Pharmacia Biotech, Switzerland) were used for the chromatography column. Semi-preparative HPLC was performed on a DIONEX Ultimate 3000 system equipped with a diode array detector and a C18 column (250 mm × 10 mm, 5 μm, YMC Co. Ltd., Kyoto, Japan).

### 3.2. Plant Material

Theroot and rhizome of *C. robustum* Maximwere collected in May 2015 from Yi Chang City, Hubei Province, China. They were identified by Dr. Xinqiao Liu from the College of Pharmacy at South-Central University for Nationalities, China. A voucher specimen (No. CR-20150501) was deposited at the herbarium of the College of Pharmacy, South-Central University for Nationalities, China.

### 3.3. Extraction and Isolation

The dried root and rhizome of *C. robustum* (30 kg) was extracted with 95% (120 L) aqueous EtOH. After concentration, the EtOH extract (6 kg) was suspended in H_2_O and then partitioned successively with petroleum ether, EtOAc, and *n*-butyl alcohol to give petroleum ether (A, 200 g), EtOAc (B, 415 g), and n-butyl alcohol (C, 2 kg) fractions, respectively. Fraction B was chromatographed over silica gel (4 kg), eluting with EtOAc in petroleum ether (0–100%, stepwise), yielding seven fractions (Fr1–Fr7). Fraction Fr4 (30 g) was chromatographed over a silica gel column eluting with CH_2_Cl_2_-MeOH (0–100%, stepwise), yielding five fractions (Fr4a1–Fr4a5). Sub-fractions Fr4a4 were separated on Sephadex LH-20 (MeOH:CHCl_3_ = 1:1) to give three fractions (Fr4a4-1, Fr4a4-2, and Fr4a4-3), respectively. Fr4a4-2 was further purified by YMC Prep-HPLC chromatography using acetonitrile-H_2_O (80%) as eluent to afford compound **1** (26 mg, Rt = 11.67 min), **2** (10 mg, Rt = 13.83 min), and **3** (45 mg, Rt = 15.68 min). Fr4a4-3 was further purified by YMC Prep-HPLC chromatography using acetonitrile-H_2_O (80%) as eluent to afford compound **4** (37 mg, Rt = 12.93 min), **5** (40 mg, Rt = 19.02 min), and **6** (19 mg, Rt = 26.71 min). Fraction C was chromatographed on a column of highly porous polymer Diaion HP-20 (5 L) and eluted with H_2_O and EtOH, successively, to give three fractions (Fc1–Fc3). Fraction c1 (160 g) was chromatographed over silica gel, eluting with MeOH in CH_2_Cl_2_ (0–100%, stepwise), to provide eight sub-fractions (Fc1a–Fc1h). Sub-fraction Fc1a (50 g) was separated by YMC RP chromatography using MeOH-H_2_O (50%) as eluent to obtain three sub-fractions (Fc1a-1–Fc1a-3), Fc1a-2 (5 g) was separated by YMC Prep-HPLC chromatography using MeOH-H_2_O (55%) as eluent to obtain compound **7** (65 mg, Rt = 14.24 min) and **8** (15 mg, Rt = 10.92 min). Sub-fraction Fc2 (95 g) was purified by YMC RP chromatography, eluting with MeOH-H_2_O (1:1), to give three fractions (Fc2a–Fc2c). Fc2a was separated by YMC Prep-HPLC chromatography using MeOH-H_2_O (39%) as eluent to obtain compound **9** (38 mg, Rt = 13.23 min). Sub-fraction Fc3 (65 g) was purified by YMC RP chromatography, eluting with MeOH-H_2_O (35%) to give four fractions (Fc3a–Fc3d). Fc2c was separated by YMC Prep-HPLC chromatography using MeOH-H_2_O (40%) as eluent to obtain compound **10** (14 mg, Rt = 33.20 min).

### 3.4. Cell Culture and CCK-8 Cell Viability Assay

RAW264.7 mouse macrophages were purchased from the Bio-Swamp life science lab (Bio-Swamp, MD, USA) and cultured in DMEM supplemented with 10% FBS, 100 U/mL of penicillin, and 100 μg/mL of streptomycin at 37 °C in 5% CO_2_. The subculture was carried out at 2 to 3 day intervals. When the cells were approximately 80% confluent, they were seeded in 96-well culture plates at 5 × 10^3^ cells per well and incubated for 24 h for adhesion. After cells had been incubated with these treatments for 24 h, the effect of compounds **1** and **3** on cell viability was analyzed. RAW264.7 cells were treated in the absence or presence of compounds **1** and **3** (5, 15, and 25 μM) for 24 h. Cell viability was determined by CCK-8 assay (data not shown).

### 3.5. Measurement of Nitric Oxide Production

LPS and Dexamethasone were purchased from Sigma Aldrich (Saint Louis, MO, USA). NO production was determined by the Griess reaction, which measures the accumulation of nitrite in the culture medium. When the cells were approximately 80% confluent, they were seeded in 96-well culture plates at 1 × 10^5^ cells per well and incubated for 24 h for adhesion. The cells were then pretreated with phenol red-free medium containing the control (0.5% DMSO) or the indicated concentrations of compounds (**1**–**10**) for 2 h and then exposed to 5 μg/mL LPS for 24 h. The supernatant (50 μL) was collected, mixed with an equal volume (50 μL) of Griess reagent I and II. The absorbance at 540 nm was measured with a microplate reader. Sodium nitrite was used to generate a standard reference curve.

### 3.6. Western Blotting Analysis

Western blotting was performed as previously described with small modifications. RAW264.7 cells were lysed in RIPA lysis buffer containing protease inhibitor cocktail (Roche Diagnostics, Mannheim, Germany). Equal amounts of protein were separated by 10% SDS polyacrylamide gel electrophoresis (SDS-PAGE) and then transferred to PVDF membrane. The membrane was then blocked with 5% non-fat dry milk in TBST (20 mM Tris-HCl, 150 mMNaCl, and 0.05% Tween-20). After incubation with a primary antibody at 4 °C overnight, the membrane was hybridized with HRP-conjugated secondary antibody for 1 h, washed three times with TBST. The signal was detected by BCIP/NBT Alkaline Phosphatase Color Development Kit (Beyotime, Haimen, China). The molecular mass of bands was verified using a Multicolor Protein Marker (Beyotime, Haimen, China).

### 3.7. Statistical Analysis

The data were expressed as the mean ± standard deviation (SD) of at least three independent experiments. To compare three or more groups, one-way analysis of variance (ANOVA) followed by the Newman−Keuls post hoc test was used. A *p*-value of less than 0.05 was considered statistically significant. Statistical analysis was performed using GraphPad Prism software, version 5.00 (GraphPad Software Inc., La Jolla, CA, USA).

### 3.8. Experimental Data of Identified Compounds

Compound *23-hydroxy-3,19-dioxo-olean-12-en-28-oic acid*. (**1**) White amorphous powder. ${\left[\alpha \right]}_{D}^{25}$ = +48° (c = 0.01, CHCl_3_), IR (KBr) ν_max_ 3533, 3392, 2962, 1702, 1672, 1386, 1262, 1037, 1000 cm^−1^, HR-ESI-MS: *m*/*z*: 483.3117 [M − H]^−^ (calculated for C_30_H_43_O_5_, 483.3110), ^1^H-NMR (CD_3_OD, 600 MHz) and ^13^C-NMR (CD_3_OD, 150 MHz) (see Table 1).

Compound *23-hydroxy-3,11-dioxo-olean-12-en-28-oic acid*. (**2**) White amorphous powder. ${\left[\alpha \right]}_{D}^{25}$ = +58° (c = 0.01, CHCl_3_), IR (KBr) ν_max_ 3477, 3264, 2972, 2871, 1708, 1686, 1460, 1383, 1180, 1159 cm^−1^, HR-ESI-MS: *m*/*z*: 483.3118 [M − H]^−^ (calculated for C_30_H_43_O_5_, 484.3110), ^1^H-NMR (CDCl_3_, 600 MHz) and ^13^C-NMR (CDCl_3_, 150 MHz) (see Table 2).

Compound *16α,23-dihydroxy-3-oxo-olean-12-en-28-oic acid*. (**3**) White amorphous powder. ${\left[\alpha \right]}_{D}^{25}$ = +60° (c = 0.01, CHCl_3_), IR (KBr) ν_max_ 3394, 2940, 1727, 1698, 1458, 1388, 1253, 1233, 1182, 1058, 1031 cm^−1^, HR-ESI-MS: *m*/*z*: 485.3271 [M + H]^−^ (calculated for C_30_H_45_O_5_, 485.3267), ^1^H-NMR (CD_3_OD, 600 MHz) and ^13^C-NMR (CD_3_OD, 150 MHz) (see Table 3).

All spectra can be found at the supplementary materials.

## 4. Conclusions

In conclusion, three new triterpene derivatives (**1**–**3**) isolated from *C. robustum* Maxim, a traditional medicine used for arthritis in the tujia ethnic areas in China, were demonstrated to have in vitro anti-inflammatory properties individually. The underlying mechanism may be associated with the regulation ofiNOS and NF-κB. This is the first report on the anti-inflammatory activity of the two triterpene (**1** and **3**) derivatives through iNOS and NF-κB. The present findings provide evidence for *C. robustum* Maxim as a promising treatment for inflammatory-related diseases.

## Figures and Tables

**Table 1:** ^1^H- and ^13^C-NMR spectral data and HMBC correlations of **1** (^1^H-NMR, 600 MHz,^13^C-NMR, 150 MHz, in CD_3_OD, *δ* ppm, *J* Hz).

Position	*δ*_C_	*δ*_H_	HMBC
1	38.8	1.84, 1.43, m	C-3, 25
2	36.7	2.31, 2.44, m	
3	219.8	—	
4	53.6	—	
5	42.7	overlapping	
6	20.6	1.39, m	
7	33.2	1.29, 1.57, m	C-26
8	43.1	—	
9	47.7	1.73, m	C-25, C-26
10	37.6	—	
11	24.8	1.97, m	
12	125.1	5.41, t, *J* = 8 Hz	C-9,18
13	143.1	—	
14	40.7	—	
15	28.8	1.08, 1.72, m	
16	26.8	1.65, 1.78, m	C-28
17	51.2	—	
18	48.2	2.79, d, *J* = 14.0 Hz	C-19, 12
19	216.2	—	
20	46.7	—	
21	48.8	2.16, m	C-29, 30
22	47.9	1.72, m	
23	68.2	3.56, 3.28, dd, *J* = 11.5Hz	C-3, 4, 5, 24
24	17.8	0.86, s	C-3, 4, 23
25	15.8	1.05, s	C-1, 5, 9
26	18.1	0.85, s	C-7, 8, 9
27	26.3	1.15, s	C-13
28	178.2	—	
29	25.2	0.95, s	C-19, 20, 30
30	25.7	1.21, s	C-19, 20, 29

**Table 2:** ^1^H- and ^13^C-NMR spectral data and HMBC correlations of **2** (^1^H-NMR, 600 MHz,^13^C-NMR, 150 MHz, in CDCl_3_, *δ* ppm, *J* Hz).

Position	*δ*_C_	*δ*_H_	HMBC
1	39.2	3.07, 1.34, m	C-3, 25
2	35.4	2.70, 2.24, m	
3	218.7	—	
4	53.0	—	
5	49.2	1.62, m	
6	18.3	1.44, m	
7	32.0	1.73, 1.65, m	C-26
8	43.8	—	
9	60.9	2.43, s	C-11, 25, 26
10	36.8	—	
11	200.0	—	
12	127.9	5.64, s	C-11, C-13, C-18
13	169.4	—	
14	45.3	—	
15	27.9	1.69, 1.25, m	
16	22.8	2.03, 1.72, m	C-28
17	46.2	—	
18	41.7	2.96, dd, *J* = 14.5, 4.5 Hz	C-12
19	44.6	1.61, 1.19, m	C-29, 30
20	30.8	—	
21	31.7	1.75, 1.65, m	C-29, 30
22	33.8	1.36, 1.25, m	
23	66.7	3.67, 3.35, dd, *J* = 11.5 Hz	C-3, 4, 24
24	17.0	0.96, s	C-3, 4, 23
25	15.8	1.30, s	C-1, 5, 9
26	19.4	0.95, s	C-7, 8, 9
27	23.7	1.35, s	C-13
28	182.6	—	
29	33.0	0.90, s	C-19, 21, 30
30	23.5	0.91, s	C-19, 21, 29

**Table 3:** ^1^H- and ^13^C-NMRspectral data and HMBC correlations of **3** (^1^H-NMR, 600 MHz,^13^C-NMR, 150 MHz, in CD_3_OD, *δ* ppm, *J* Hz).

Position	*δ*_C_	*δ*_H_	HMBC
1	38.8	1.83, 1.43, m	C-3, 25
2	36.7	2.42, 2.32, m	
3	220.0	—	
4	53.6	—	
5	48.3	1.90, m	
6	20.7	1.38, m	
7	33.5	1.59, 1.28, m	C-26
8	40.6	—	
9	46.7	1.76, m	C-11, 25, 26
10	37.6	—	
11	24.7	1.91, m	
12	123.3	5.27, t, *J =* 7.9 Hz	C-9, 14, 18
13	145.3	—	
14	43.0	—	
15	36.7	2.41, 2.32, m	
16	75.3	4.41, s	C-28
17	49.7	—	
18	42.3	2.94, dd, *J* = 14.5, 4.5 Hz	C-12
19	47.7	2.23, 0.97, m	C-29, 30
20	31.5	—	
21	36.2	1.79, 1.32, m	C-29, 30
22	32.9	1.83, 1.69, m	
23	68.1	3.53, 3.27, dd, *J =* 11.5 Hz	C-3, 5, 24
24	18.1	0.85, s	C-3, 4, 23
25	15.9	0.97, s	C-1, 5, 9
26	17.8	0.81, s	C-7, 8, 9
27	27.3	1.36, s	C-13
28	181.1	—	
29	25.3	0.91, s	C-19, 21, 30
30	33.6	0.82, s	C-19, 21, 29

## Supplementary Materials

The following are available online, Figure S1: ^1^H-NMR (600 MHz, CD_3_OD) spectrum of compound **1**, Figure S2: ^13^C-NMR (150 MHz, CD3OD) spectrum of compound **1**, Figure S3: DEPT-135° spectrum of compound **1**, Figure S4: DEPT-90° spectrum of compound **1**, Figure S5: HSQC spectrum of compound **1**, Figure S6: HMBC spectrum of compound **1**, Figure S7: HR-ESI-MS spectrum of compound **1**, Figure S8: IR (KBr disc) spectrum of compound **1**, Figure S9: ^1^H-NMR (600 MHz, CDCl_3_) spectrum of compound **2**, Figure S10: ^13^C-NMR (150 MHz, CDCl_3_) spectrum of compound **2**, Figure S11: HSQC spectrum of compound **2**, Figure S12: HMBC spectrum of compound **2**, Figure S13: HR-ESI-MS spectrum of compound **2**, Figure S14: IR (KBr disc) spectrum of compound **2**, Figure S15: ^1^H-NMR (600 MHz, CD_3_OD) spectrum of compound **3**, Figure S16: ^13^C-NMR (150 MHz, CD_3_OD) spectrum of compound **3**, Figure S17: HSQC spectrum of compound **3**, Figure S18: HMBC spectrum of compound **3**, Figure S19: ROE spectrum of compound **3**, Figure S20: HR-ESI-MS spectrum of compound **3**, Figure S21: IR (KBr disc) spectrum of compound **3**.
