# Supplementary material for: Anti-Inflammatory Triterpenoids from the Caulophyllum robustum Maximin LPS-Stimulated RAW264.7 Cells
Source: Molecules. 2018 May 11;23(5):1149. doi: 10.3390/molecules23051149 (PMC6099581; doi:10.3390/molecules23051149)
Supplement: Supplementary file 1 [file molecules-23-01149-s001.pdf]

molecules

Supplementary material

Anti-InflammatoryTriterpenoids from the *Caulophyllum robustum* Maxim in LPS-StimulatedRAW264.7 Cells

Bin-Hua Qin [1], Xin-QiaoLiu[1,*], Qiao-Yu Yuan[2], Jing Wang[1]and Hai-Yan Han [1]

1 School of Pharmaceutical Sciences, South-Central University for Nationalities, Wuhan 430074, China; binhuawuhan@163.com (B.-H.Q.); wjingwuhan@126.com (J.W.); 15827253179@163.com (H.-Y.H.)
2 Department of Bioengineering, Wuhan Polytechnic, Wuhan 430074, China; yqyscuec@163.com
* Correspondence: lxqscuec@163.com or 3095709@mail.scuec.edu.cn; Tel.: +86-27-6784-1196

Table of contents

Figure S1. ^{1}H-NMR (600 MHz, CD_3OD) spectrum of compound **1**

Figure S2. ^{13}C-NMR (150 MHz, CD_3OD) spectrum of compound **1**

Figure S3. DEPT-135° spectrum of compound **1**

Figure S4. DEPT-90° spectrum of compound **1**

Figure S5. HSQC spectrum of compound **1**

Figure S6. HMBC spectrum of compound **1**

Figure S7. HR-ESI-MS spectrum of compound **1**

Figure S8. IR (KBr disc) spectrum of compound **1**

Figure S9. ^{1}H-NMR (600 MHz, $CDCl_3$) spectrum of compound **2**

Figure S10. ^{13}C-NMR (150 MHz, $CDCl_3$) spectrum of compound **2**

Figure S11. HSQC spectrum of compound **2**
Figure S12. HMBC spectrum of compound **2**

Figure S13. HR-ESI-MS spectrum of compound **2**

Figure S14. IR (KBr disc) spectrum of compound **2**

Figure S15. ^{1}H-NMR (600 MHz, CD_3OD) spectrum of compound **3**

Figure S16. ^{13}C-NMR (150 MHz, CD_3OD) spectrum of compound **3**

Figure S17. HSQC spectrum of compound **3**

Figure S18. HMBC spectrum of compound **3**

Figure S19. ROE spectrum of compound **3**

Figure S20. HR-ESI-MS spectrum of compound **3**

Figure S21. IR (KBr disc) spectrum of compound **3**

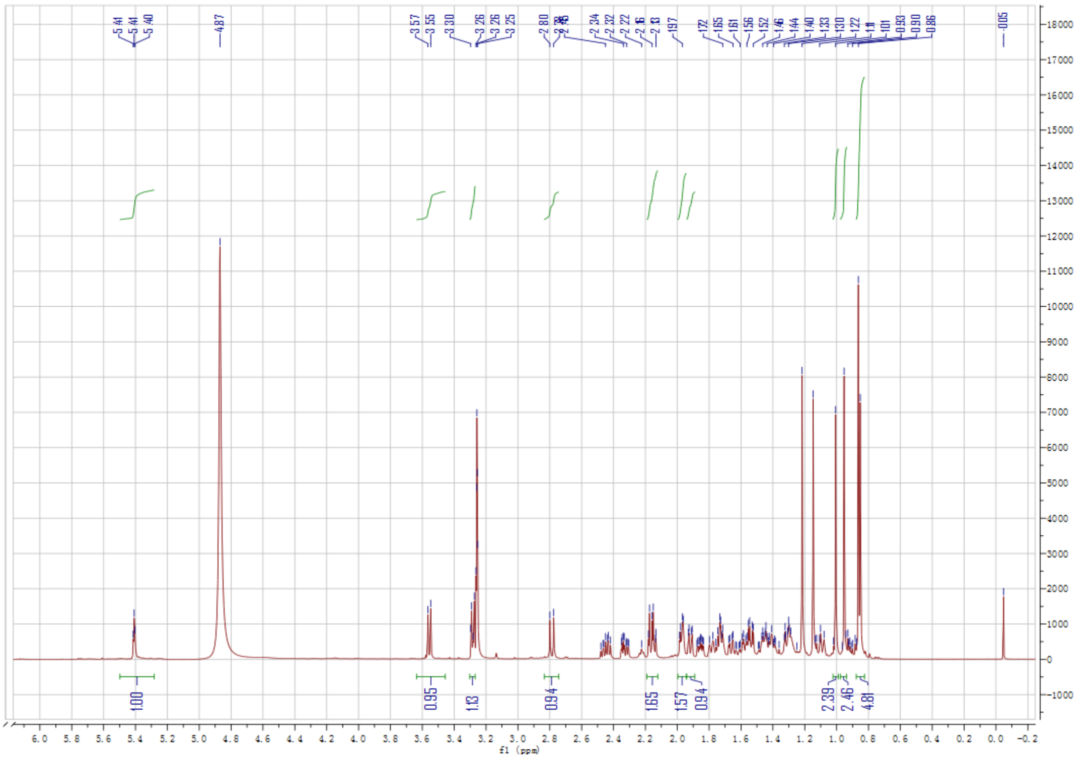

Figure S1. 1H-NMR (600 MHz, CD_3OD) spectrum of compound **1**

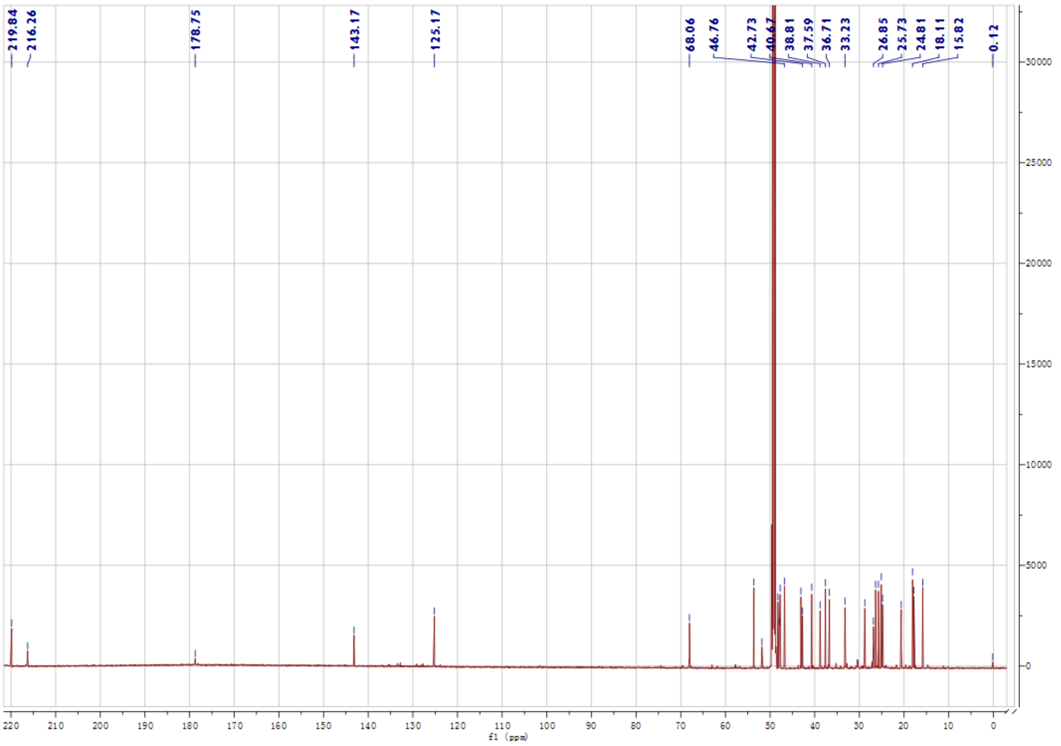

Figure S2. ^{13}C-NMR (150 MHz, CD_3OD) spectrum of compound **1**

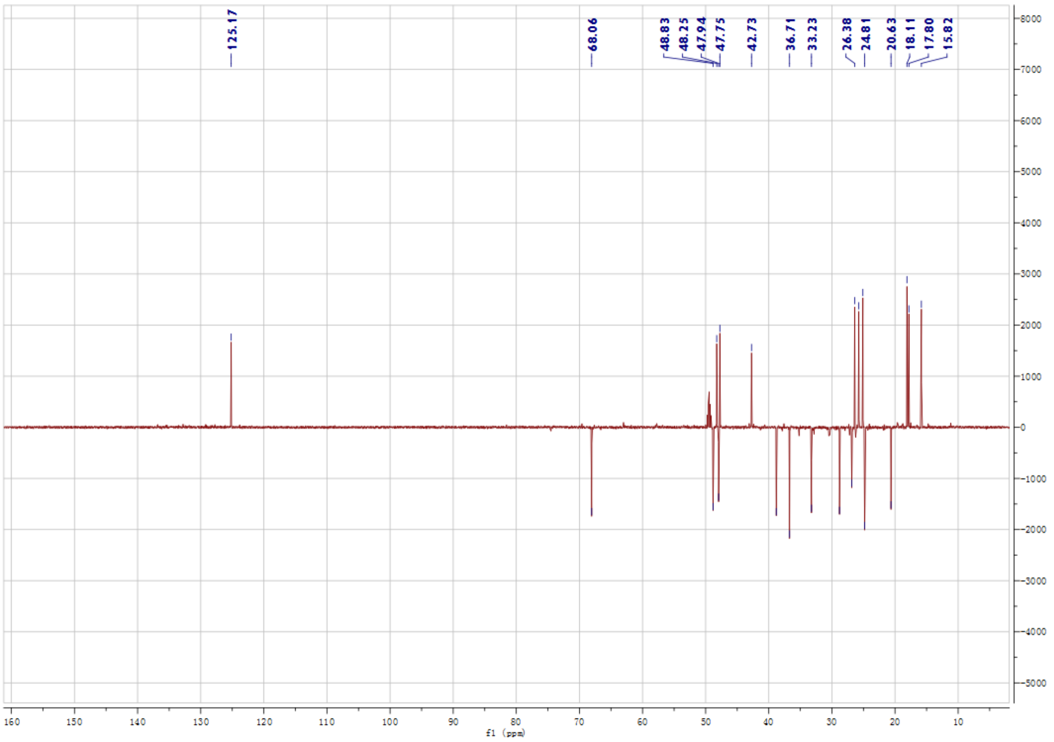

Figure S3. DEPT-135° spectrum of compound **1**

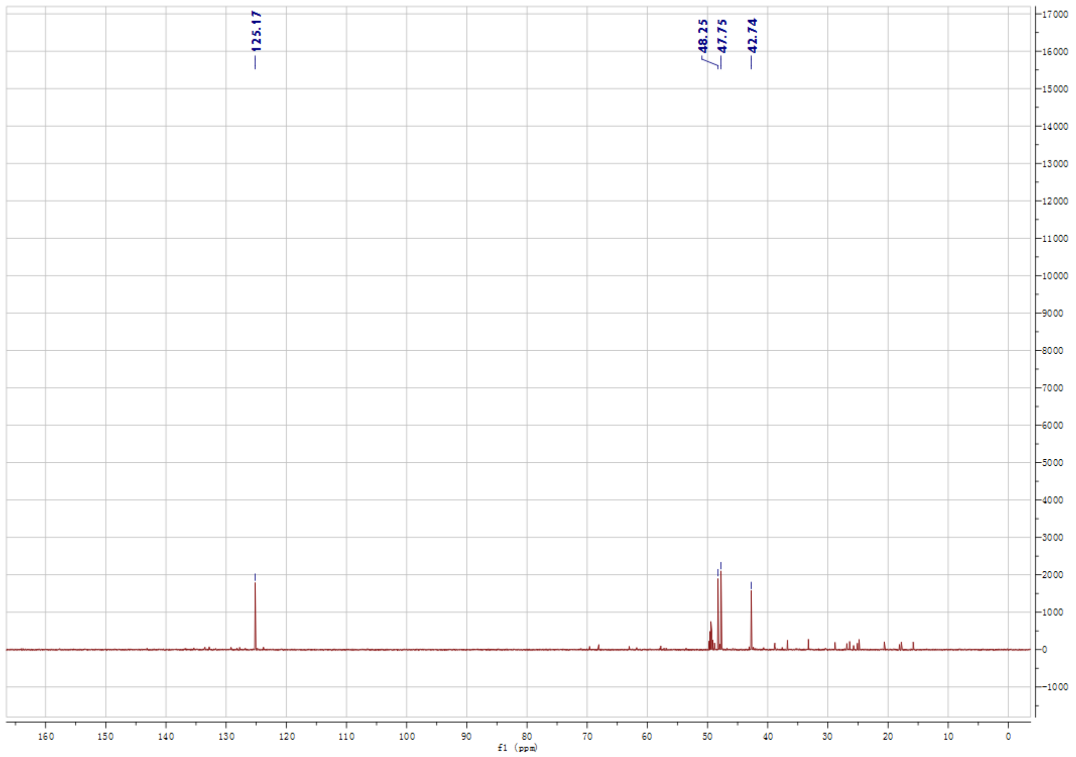

Figure S4. DEPT-90° spectrum of compound **1**

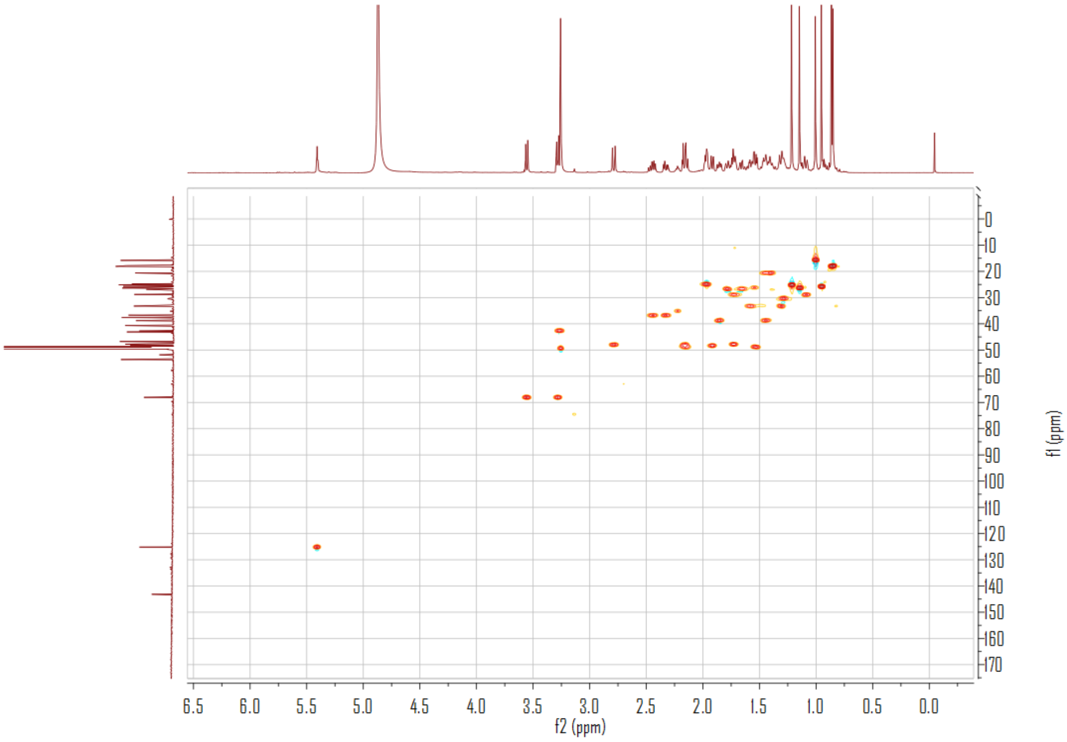

Figure S5. HSQC spectrum of compound **1**

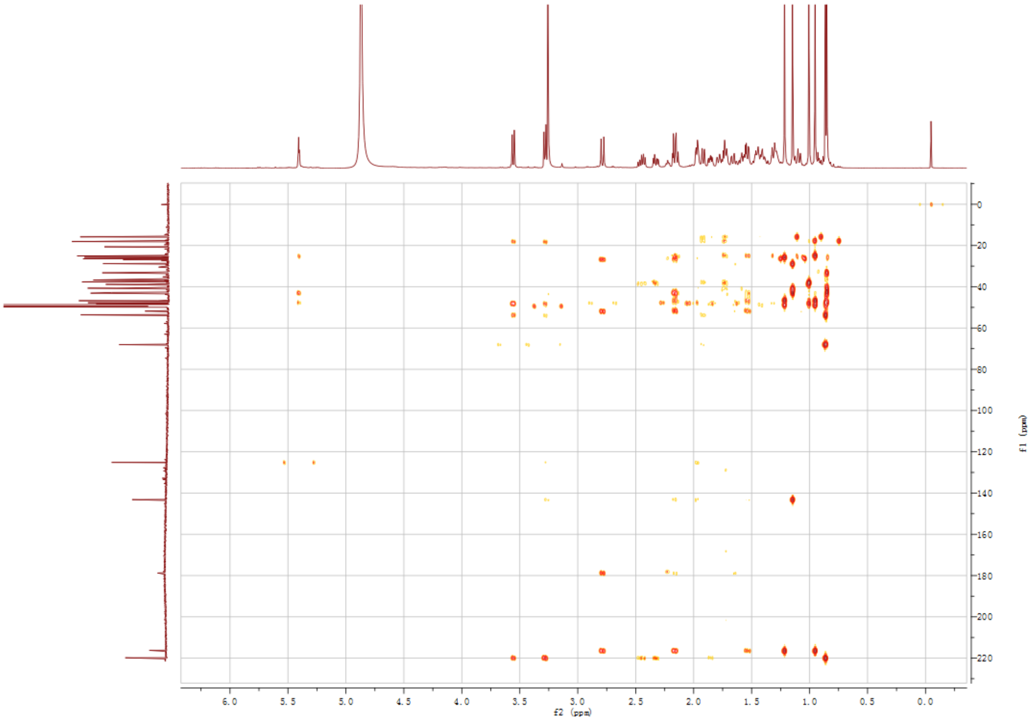

Figure S6. HMBC spectrum of compound **1**

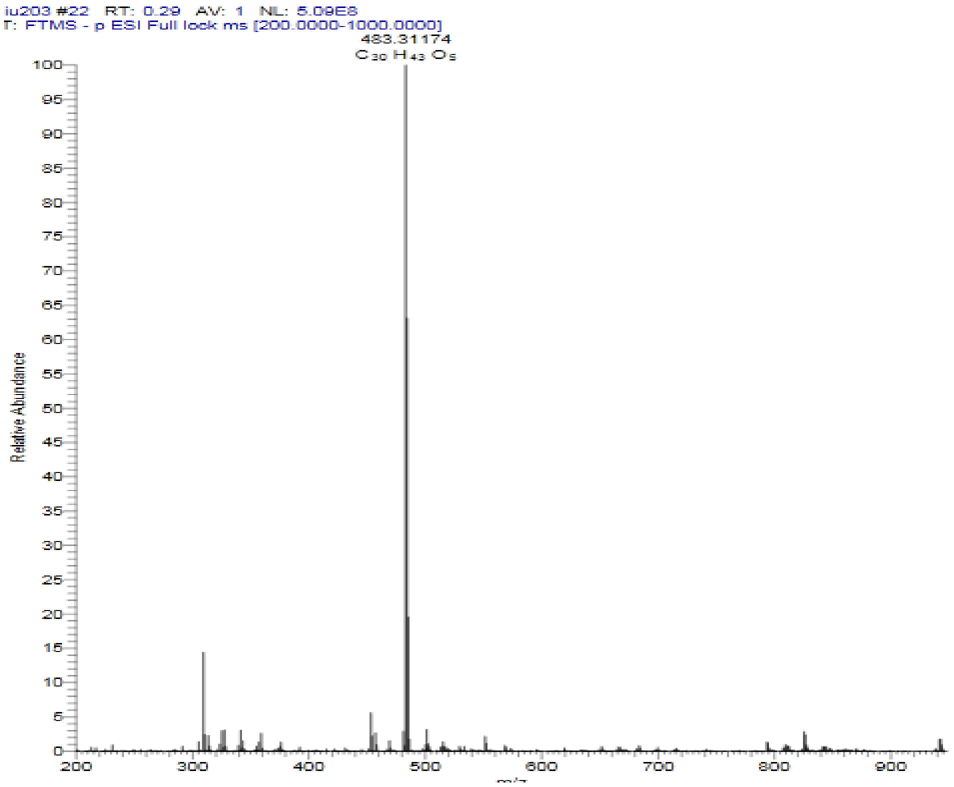

Figure S7. HR-ESI-MS spectrum of compound **1**

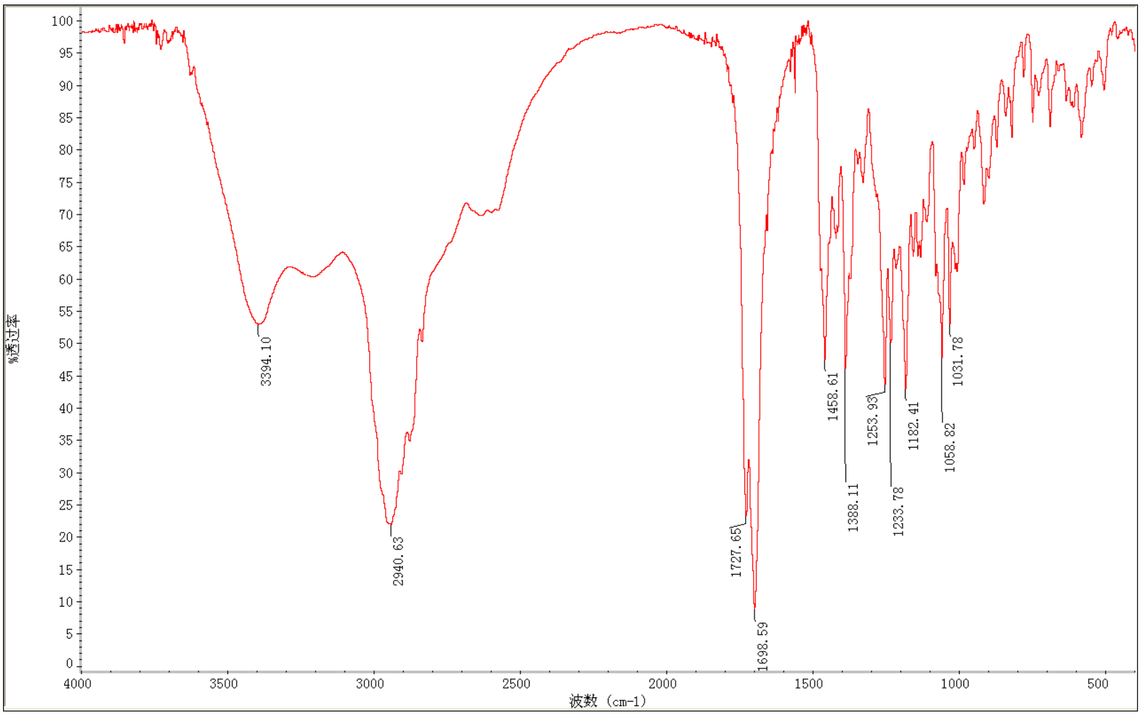

Figure S8. IR (KBr disc) spectrum of compound **1**

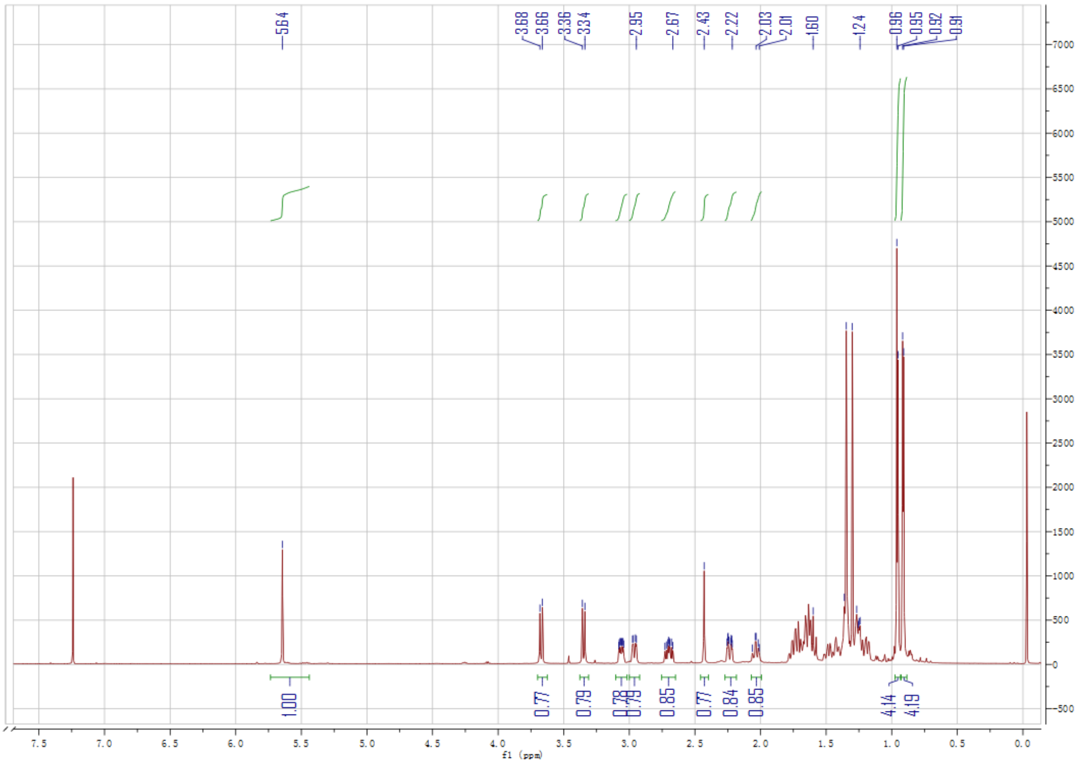

Figure S9. ^{1}H-NMR (600 MHz, $CDCl_3$) spectrum of compound **2**

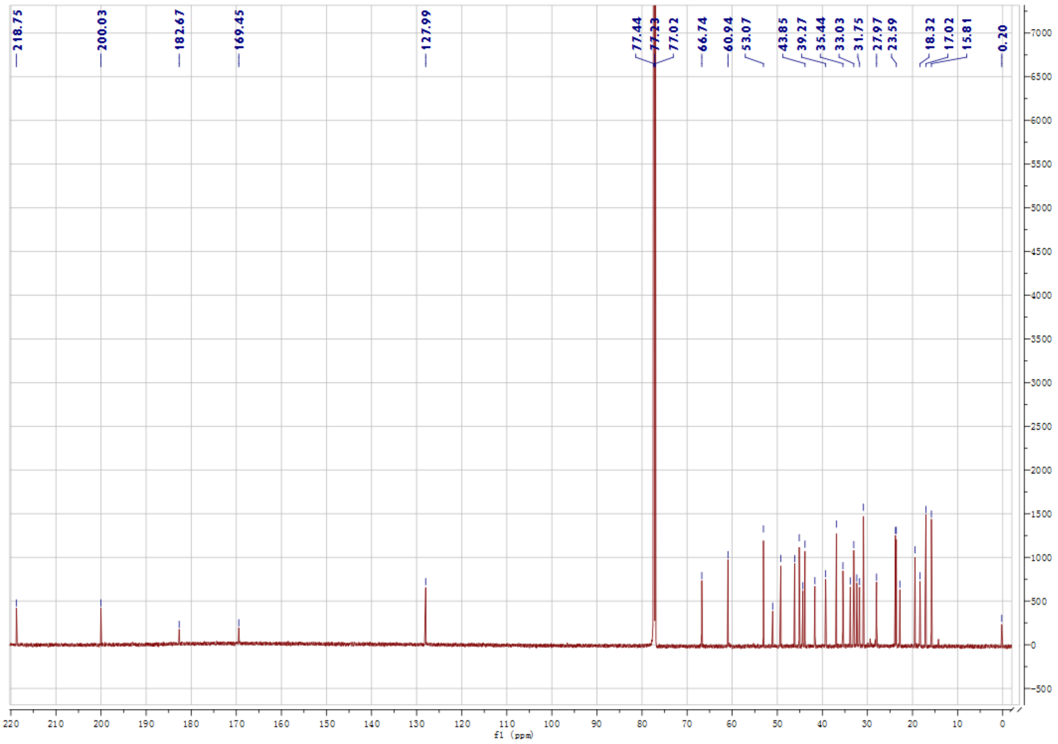

Figure S10. ^{13}C-NMR (150 MHz, $CDCl_3$) spectrum of compound **2**

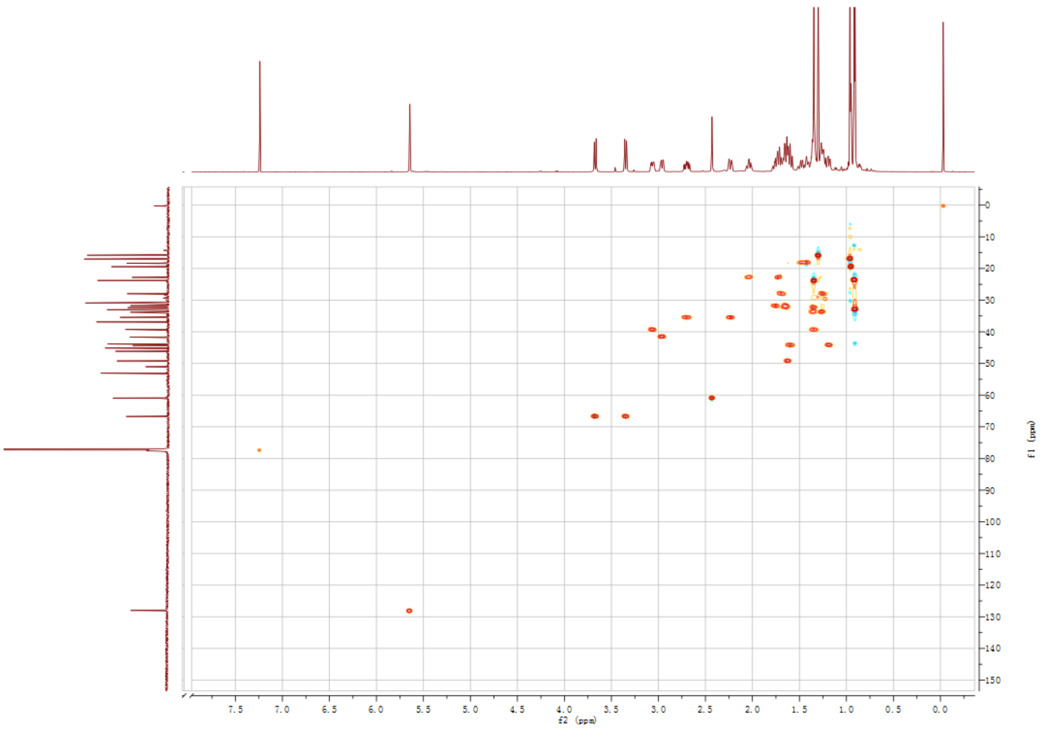

Figure S11. HSQC spectrum of compound **2**

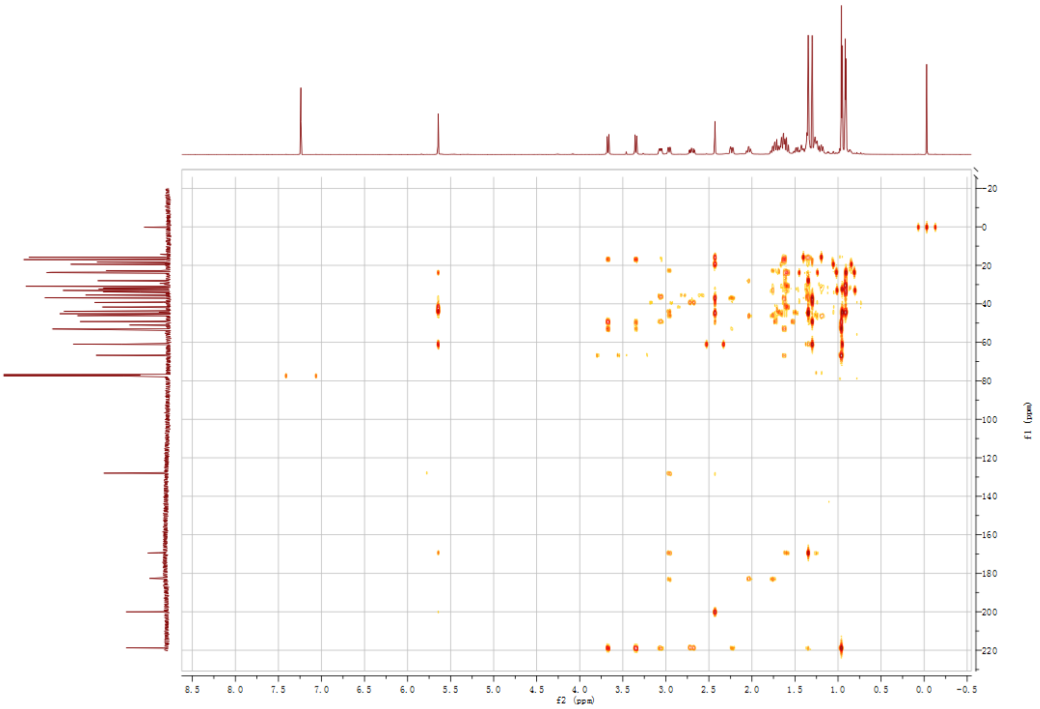

Figure S12. HMBC spectrum of compound **2**

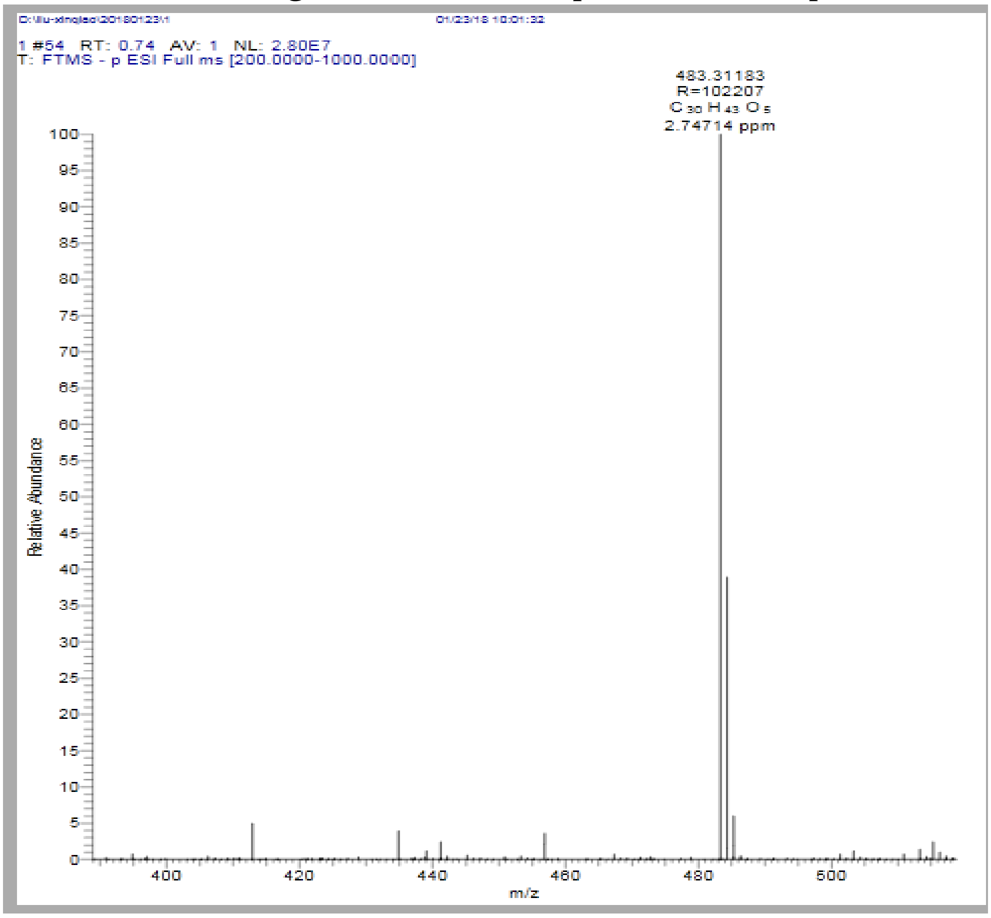

Figure S13. HR-ESI-MS spectrum of compound **2**

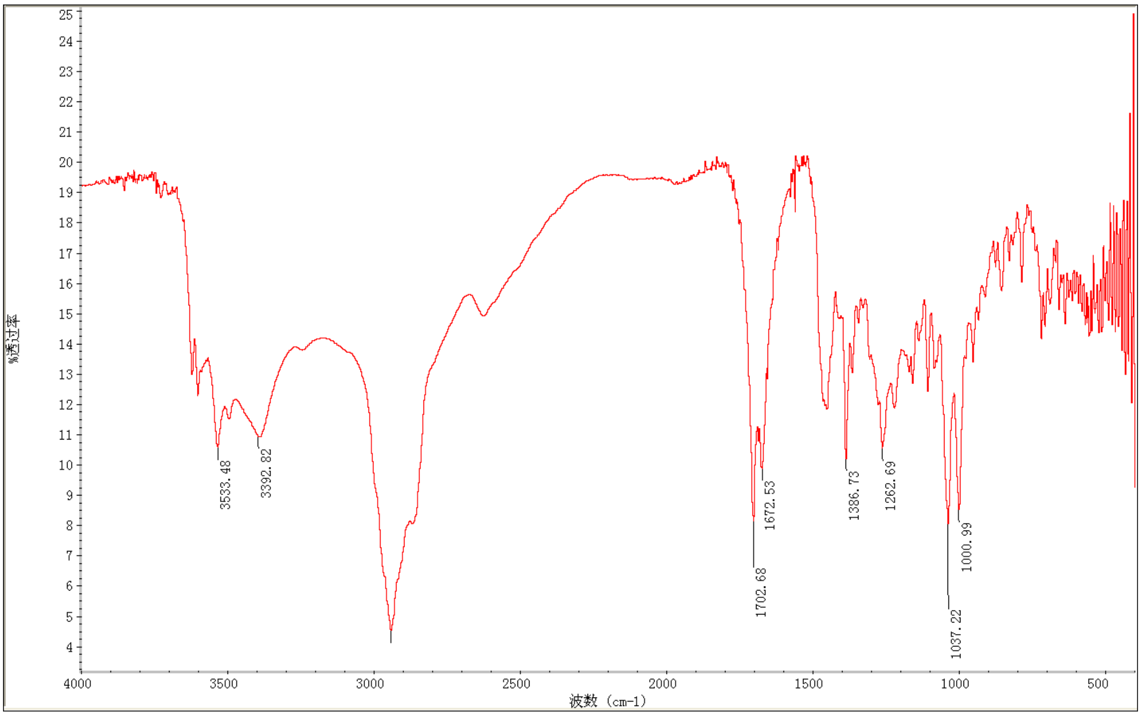

Figure S14. IR (KBr disc) spectrum of compound **2**

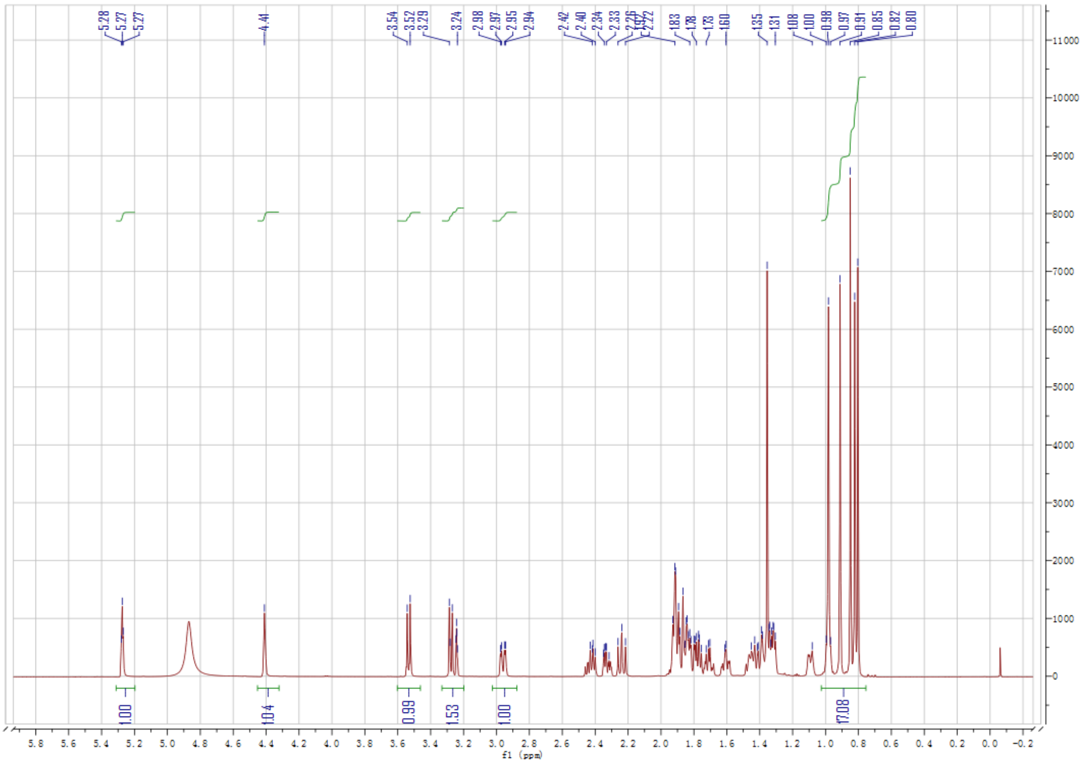

Figure S15. ^{1}H-NMR (600 MHz, CD_3OD) spectrum of compound **3**

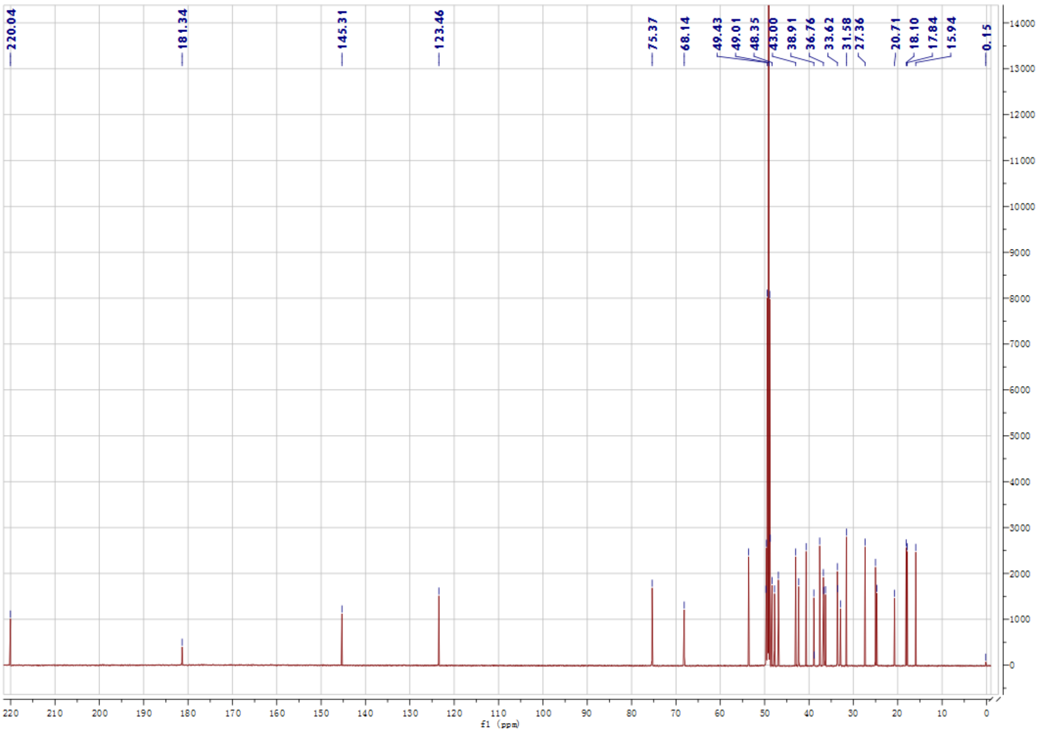

Figure S16. ^{13}C-NMR (150 MHz, CD_3OD) spectrum of compound **3**

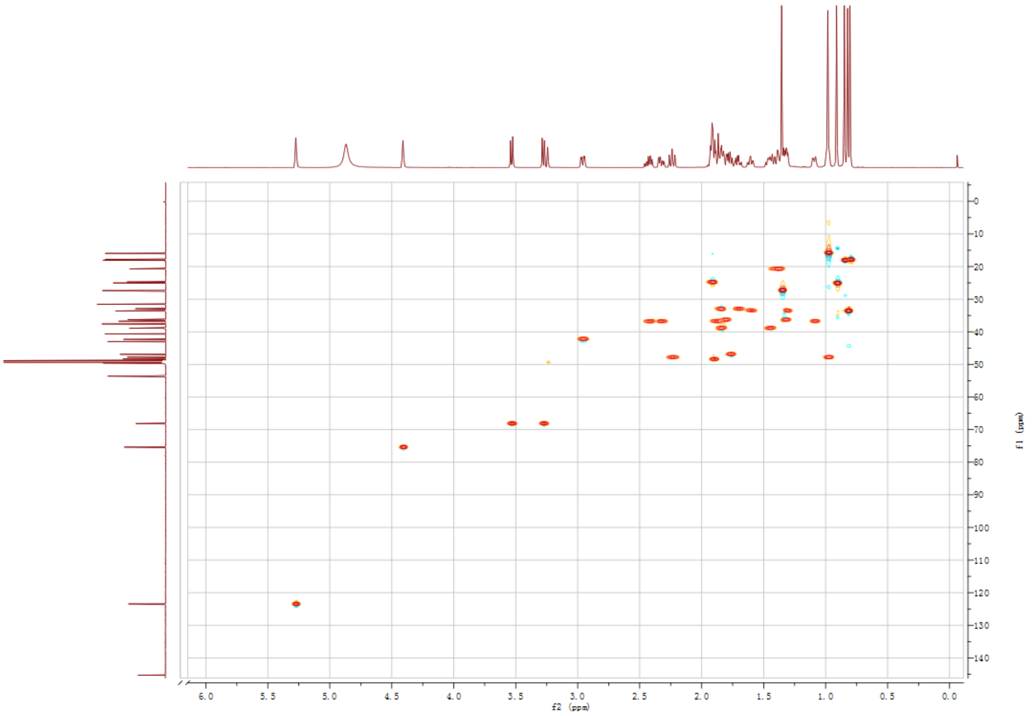

Figure S17. HSQC spectrum of compound **3**

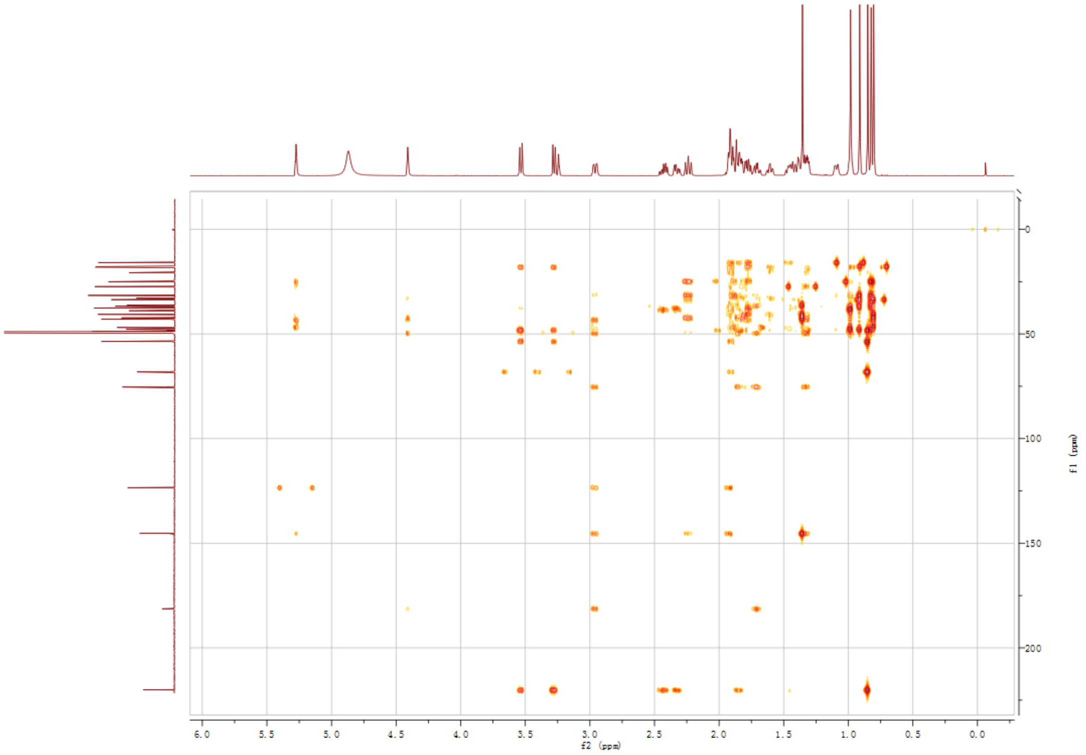

Figure S18. HMBC spectrum of compound **3**

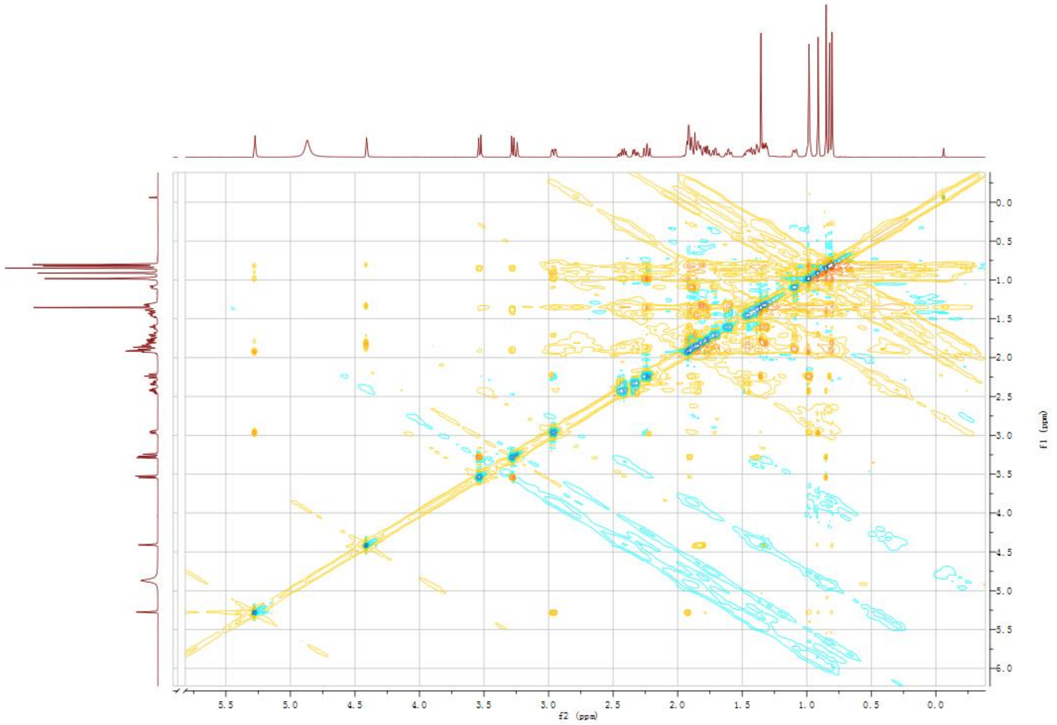

Figure S19. ROE spectrum of compound **3**

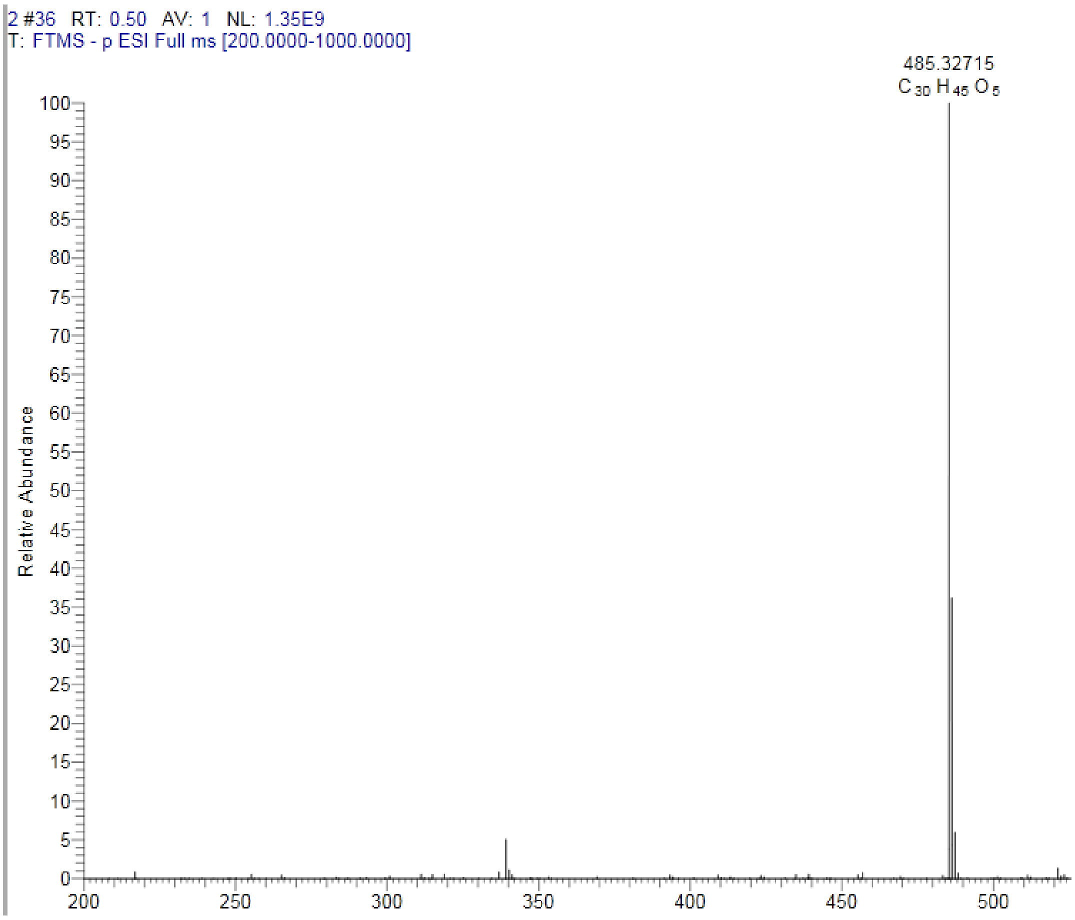

Figure S20. HR-ESI-MS spectrum of compound **3**

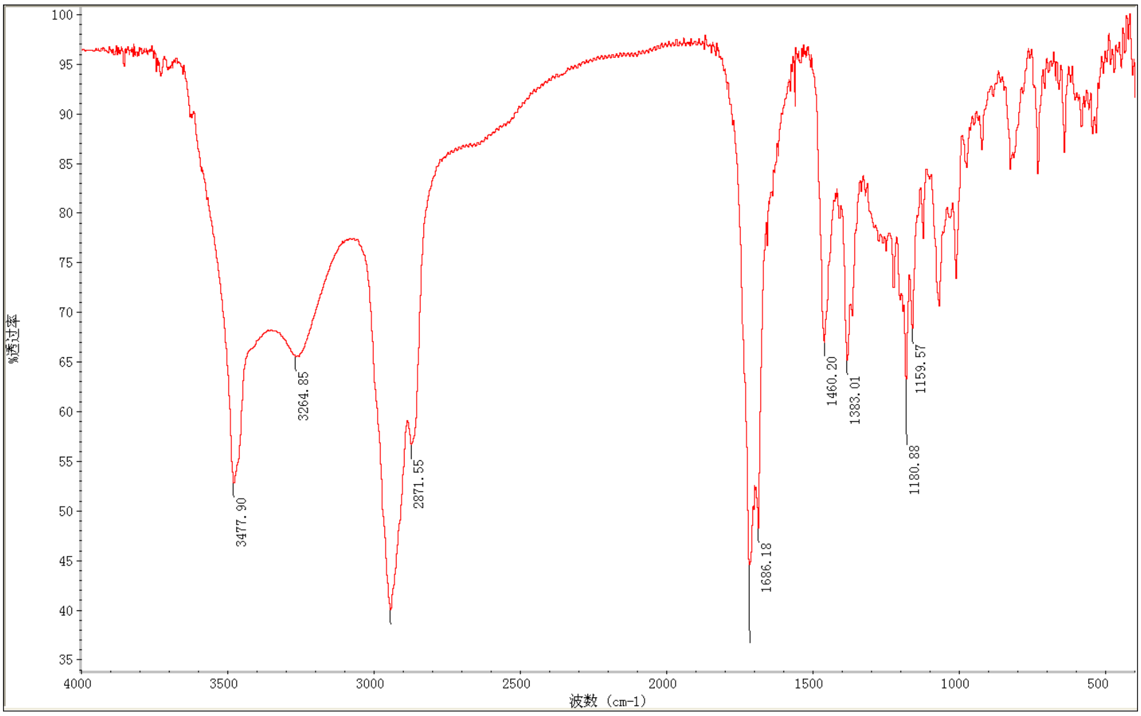

Figure S21. IR (KBr disc) spectrum of compound **3**
